# In-Patient Hospital Treatment for Insured Persons

**Published:** 1912-12-07

**Authors:** 


### THE VOLUNTARY HOSPITALS AND PARLIAMENT.

### In-patient Hospital Treatment for Insured Persons.

On November 27 Mr. Cooper, Sir John Lonsdale, and Sir C. Kinloch-Cooke questioned the Government on the subject of hospital treatment for insured persons. We give the verbatim report of these questions, and Mr. Masterman's answers, with a correction of the error into which Mr. Masterman fell as to the average number of in-patients present in the hospitals at any one time. It will be seen Mr. Masterman intimated that "business arrangements can be entered into between hospitals and approved societies in the interest of insured persons under the Act." "The societies would arrange, as some do at present, to contribute a certain amount to a hospital in return for a certain number of beds being available for their members." It will thus be seen that the Government have given their approval to the *pro rata* plan The Hospital has urged upon voluntary hospitals as a solution of the difficulty, without infringing the principles of the voluntary system.

### The Questions in the House of Commons.

Mr. Cooper asked the Chancellor of the Exchequer if he will state what arrangements he has made for insured persons requiring surgical and other treatment which can only be adequately given in hospitals and similar institutions, seeing that insured persons are no longer eligible for hospital treatment as necessitous persons?

Sir John Lonsdale asked the Chancellor of the Exchequer if he is aware that it is estimated that of the insured persons who will claim medical benefit after the 15th January next at least 30,000 a week will need for their adequate medical treatment hospital provision; and if he will state what provision will be made for these persons in the event of their being unable or unwilling to secure treatment in the existing charitable institutions?

Mr. Masterman: These questions would appear to be based on a misapprehension. Surgical and other cases which cannot, consistently with the interests of the patient, be properly undertaken by a practitioner on the panel set up under Section 15, are not affected by the Act, and there would appear to be no reason why such cases should be less eligible in the future than in the past for treatment in hospitals and similar institutions. I may state that the average number of in-patients present in the hospitals at any one time would appear to be only a small fraction of the number suggested in the question.

### A Misapprehension.

[This reply is evidently based on misapprehension. Every such case is affected, for (*a*) the Act disqualifies these cases for admission to a voluntary hospital, and (*b*) affords no other hospital provision for such cases. The last part of the answer is not the fact. There were 821 voluntary hospitals in 1910, of 172 of which we have the figures. These 172 had under treatment as in-patients during every day throughout 1910 22,000 persons. Some 50,000 in-patients probably were treated every day in the voluntary hospitals alone in 1910.—Ed. The Hospital.]

Sir J. Lonsdale: May I ask the right hon. gentleman whether he is aware that the income of the voluntary hospitals has suffered severely in consequence of the withdrawal of subscriptions, and whether he does not think it necessary to enter into business arrangements with hospitals for the treatment of insured persons?

Mr. Masterman: Business arrangements can be entered into between hospitals and approved societies in the interest of insured persons under the Act. I have no information as to any decrease of voluntary subscriptions in consequence of the Act.

Sir C. Kinloch-Cooke: Would not the funds of the approved societies go down in that case?

Mr. Masterman: The societies would arrange, as some do at present, to contribute a certain amount to a hospital in return for a certain number of beds being available for their members.

### Pro Rata Payments.

Accepting the view of the Government as expressed in the last two answers of Mr. Masterman quoted above, it implies that the *pro rata* system could be adopted under the Act. It may so be desirable for each voluntary hospital to ascertain what it could attempt in the way of providing beds for insured persons on a *pro rata* basis should opportunity offer.

